# Tackling Virulence of *Pseudomonas aeruginosa* by the Natural Furanone Sotolon

**DOI:** 10.3390/antibiotics10070871

**Published:** 2021-07-17

**Authors:** Mohammed F. Aldawsari, El-Sayed Khafagy, Ahmed Al Saqr, Ahmed Alalaiwe, Hisham A. Abbas, Moataz A. Shaldam, Wael A. H. Hegazy, Reham M. Goda

**Affiliations:** 1 Department of Pharmaceutics, College of Pharmacy, Prince Sattam Bin Abdulaziz University, Al-kharj 11942, Saudi Arabia; moh.aldawsari@psau.edu.sa (M.F.A.); a.alsaqr@psau.edu.sa (A.A.S.); a.alalaiwe@psau.edu.sa (A.A.); 2 Department of Pharmaceutics and Industrial Pharmacy, Faculty of Pharmacy, Suez Canal University, Ismailia 41522, Egypt; 3 Department of Microbiology and Immunology, Faculty of Pharmacy, Zagazig University, Zagazig 44519, Egypt; hishamabbas2008@gmail.com (H.A.A.); waelmhegazy@daad-alumni.de (W.A.H.H.); 4 Department of Pharmaceutical Chemistry, Faculty of Pharmacy, Kafrelsheikh University, Kafr El-Sheikh 33511, Egypt; dr_moutaz_986@yahoo.com; 5 Department of Microbiology and Biotechnology, Faculty of Pharmacy, Delta University for Science and Biotechnology, Gamasa 35712, Egypt; Dr_reham_magdy@yahoo.com

**Keywords:** sotolon, antibiotic resistance, natural furanone, *Pseudomonas aeruginosa*, virulence inhibition

## Abstract

The bacterial resistance development due to the incessant administration of antibiotics has led to difficulty in their treatment. Natural adjuvant compounds can be co-administered to hinder the pathogenesis of resistant bacteria. Sotolon is the prevailing aromatic compound that gives fenugreek its typical smell. In the current work, the anti-virulence activities of sotolon on *Pseudomonas aeruginosa* have been evaluated. *P. aeruginosa* has been treated with sotolon at sub-minimum inhibitory concentration (MIC), and production of biofilm and other virulence factors were assessed. Moreover, the anti-quorum sensing (QS) activity of sotolon was in-silico evaluated by evaluating the affinity of sotolon to bind to QS receptors, and the expression of QS genes was measured in the presence of sotolon sub-MIC. Furthermore, the sotolon in-vivo capability to protect mice against *P. aeruginosa* was assessed. Significantly, sotolon decreased the production of bacterial biofilm and virulence factors, the expression of QS genes, and protected mice from *P. aeruginosa*. Conclusively, the plant natural substance sotolon attenuated the pathogenicity of *P. aeruginosa,* locating it as a plausible potential therapeutic agent for the treatment of its infections. Sotolon can be used in the treatment of bacterial infections as an alternative or adjuvant to antibiotics to combat their high resistance to antibiotics.

## 1. Introduction

*Pseudomonas aeruginosa* is a leading gram-negative opportunistic human nosocomial pathogen that mainly infects immunocompromised patients [1,2,3]. *P. aeruginosa* is a recognized ubiquitous human pathogen and has been listed on the ESKAPE serious pathogen list (*Enterococcus faecium*, *Staphylococcus aureus*, *Klebsiella pneumoniae*, *Acinetobacter baumannii*, *Pseudomonas aeruginosa*, and *Enterobacter* spp.) [4]. It is reported to be the second most frequently isolated pathogen from intensive care unit patients, and is mainly transmitted through hospital co-workers and colonized patients [5]. Virtually, *P. aeruginosa* can infect all body tissues and causes a variety of acute and chronic infections, such as urinary tract, respiratory and eye infections, meningitis, and wound sepsis as reviewed [2,6,7].

Quorum sensing (QS) is the regulation of gene expression in response to cell-population density variations. QS bacterial systems utilize chemical signal molecules called autoinducers that bind to their QS receptors. Consequently, diverse virulence factors are regulated to enable the establishment of bacterial infections [2,8,9,10]. Importantly, QS plays a crucial role in regulation of bacterial biofilms, which results in enhancement of both bacterial pathogenesis and resistance to antimicrobials [2,8,11,12,13]. As such, inhibition of biofilm formation has a great impact on reduction of bacterial resistance [2,11,14]. *P. aeruginosa* is known to establish persistent biofilms on indwelling medical devices [15]. It employs an efficient collection of enzymes and other virulence factors to attack and infect a wide diversity of human tissues [16]. The persistence of *P. aeruginosa* infections can be attributed to its ability to produce many extracellular virulence factors such as pyocyanin, rhamnolipid, alginate, exotoxin A, elastase, LasA, and alkaline protease, the expression of which is controlled by the quorum sensing (QS) system [17,18]. QS is a signaling system that orchestrates the communications among *P. aeruginosa* cells; it utilizes small diffusible molecules including 4-quinolones [19] and N-acylhomoserine lactones as butanoyl homoserine lactone (C4-HSL), and dodecanoyl homoserin lactone (3-oxo-C12-HSL) [20]. By their roles, the signaling inducers are involved in virulence factor regulation, exoenzymes production, bacterial adhesion and the formation of biofilm [21] and in antibiotic resistance [22].

*P. aeruginosa* owns nearly all of the known antimicrobial resistance mechanisms; this explains the ineffectiveness of empirical antibiotic treatments [23]. It is intrinsically resistant to many antibiotics, due to low outer membrane permeability and adaptive resistance mechanisms [7,24,25]. Moreover, the emergence of clinical strains with modified virulence factors makes treatment difficult [26]. The current therapeutic options, such as β-lactam and aminoglycoside drugs, usually fail because of widespread antibiotic resistance [24]. To circumvent this challenge, increasing attention has been paid in recent years to anti-QS compounds as potential therapeutic agents. Anti-QS compounds, when administered along with antibiotics, have the potential to increase the pathogen’s susceptibility to antibiotics [8,27,28,29]. Moreover, targeting QS has no effect on the bacterial growth. As a result, the possibility of the emergence of resistance is much lower than in the case of the use of antibiotics [8,29,30]. The idea behind the use of QS inhibitors is targeting virulence factors with minimal to no effect on bacterial growth, which could potentially decrease the risk of bacterial resistant strains selection [12]. As a promising alternative approach to conquer the bacterial resistance, several studies have been elucidated to explore efficient QS inhibitors [8,11,13,14,30,31,32,33].

The natural products extracted from plant and algae were among the first compounds to be explored for their anti-QS activities. The first anti-QS compound, a furanone derivative, was characterized from the seaweed *Delisea pulchra*. Naturally occurring furanones such as halogenated furanones from *Delisea pulchra* have been found to inhibit bacterial infection and biofilm formation by *P. aeruginosa* [34]. In this direction, we aimed to investigate the activity of other furanone derivatives. Sotolon is a furanone derivative lactone (3-hydroxy-4,5-dimethyl-2(5H)-furanone) and is an extremely powerful aroma compound. Sotolon was first isolated from the herb fenugreek in 1975, and it is the major aroma and flavor component of fenugreek seed. It is one of numerous flavor aromatic components of artificial maple syrup, and is also present in molasses, roast tobacco, aged sake, aged rum, and white wine [35]. Sotolon is known as being the main odorant and has also been considered as a potential aging marker of these wine types. The chiral lactone of sotolon is a powerful odorant that depends on its concentration and enantiomeric distribution [35,36]. In our previous study, sotolon was found to have in-vitro anti-virulence activity against *Serratia marcescens* [37]. In the current study, we are interested to investigate the in-vitro and in-vivo effects of sotolon on QS controlled virulence factors of *P. aeruginosa*.

## 2. Results

### 2.1. Minimum Inhbitory Concentration (MIC) of Sotolon

Sotolon could prevent the *P. aeruginosa* PAO1 strain growth at 200 µg/mL. The sub-MIC concentrations selected to evaluate the anti-virulence and anti-QS activities of sotolon are 50 µg/mL and 25 µg/ mL, which correspond to 1/4 MIC and 1/8 MIC, respectively.

### 2.2. Sotolon Inhibited Biofilm Inhibition

**Figure 1:**
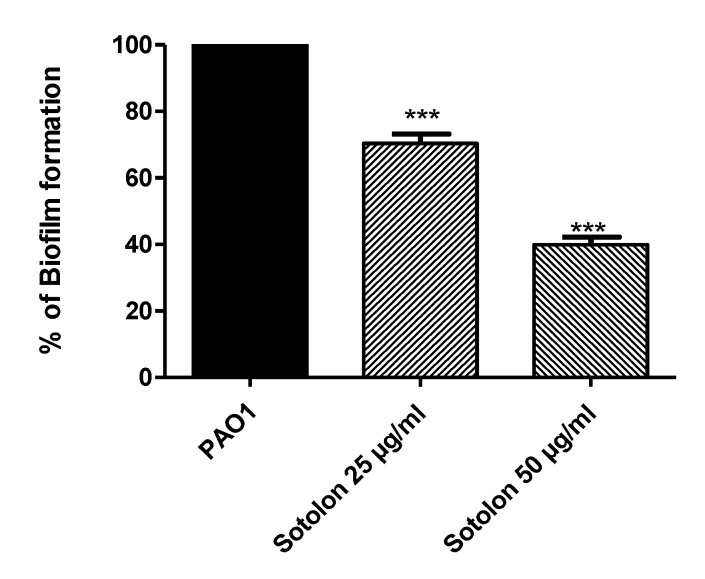
Inhibition of biofilm production of PAO1 strain by sotolon. PAO1 cultures were prepared with and without Sotolon sub-MIC (1/4 and 1/8 MIC). One-way ANOVA test (Graphpad Prism 8 software) was employed to compare between the results of sotolon sub-MIC treated and untreated PAO1 cultures. The results were assumed statistically significant when *p* values < 0.05. The data obtained were presented as mean ± standard error of percentage change from untreated cultures. Sotolon in 1/4 and 1/8 MIC significantly reduced the biofilm formation (*p* < 0.0001). *** = *p* < 0.001.

The formation of biofilm was assayed in the presence and absence of sotolon to show the sotolon ability to decrease the biofilm production. Sotolon at 1/4 and 1/8 MIC significantly reduced the production of biofilm biomass (*p* < 0.05). The biofilm inhibition percentage was about 29% at 25 µg/mL and 60% at 50 µg/ ml (Figure 1).

### 2.3. Sotolon Inhibited P. auerginosa Virulence Factors

**Figure 2:**
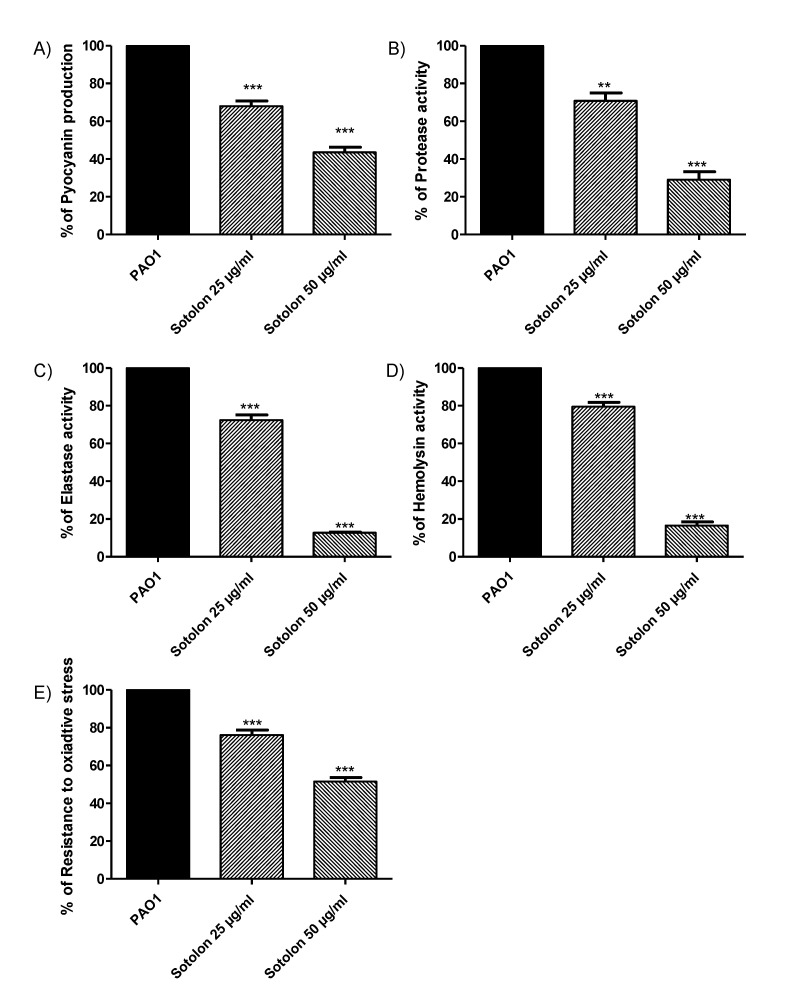
Inhibition of PAO1 virulence factors by sotolon. PAO1 cultures were prepared with and without Sotolon sub-MIC (1/4 and 1/8 MIC). One-way ANOVA test (Graphpad Prism 8 software) was utilized to compare between the results of sotolon sub-MIC treated and untreated PAO1 cultures. The results were considered statistically significant when *p* values < 0.05. The data obtained were presented as mean ± standard error of percentage change from untreated cultures. (**A**) Pyocyanin production: sotolon in sub-MIC significantly reduced the production of pyocyanin (*p* < 0.0001). (**B**) Protease production: sotolon significantly reduced the production of protease, *p* = 0.0022 and *p* < 0.0001 in 1/4 MIC and 1/8 MIC, respectively. (**C**) Elastase production: elastase was significantly reduced in the presence of sotolon in sub-MIC (*p* < 0.0001). (**D**) Hymolysins production: Sotolon significantly diminished the hemolytic activities of POA1 strain (*p* < 0.0001). (**E**) Resistance to oxidative stress: sotolon in sub-MIC significantly enhanced the sensitivity to oxidative stress (*p* < 0.0001). ** = *p* < 0.01, *** = *p* < 0.001.

Pyocyanin bluish-green pigment is a characteristic virulence factor of *P. aeruginosa*. When pyocyanin production was measured in sotolon presence and absence, it was found that sotolon lowered pyocyanin levels by about 32% at 25 µg/mL and 57% at 50 µg/mL (Figure 2A). Importantly, sotolon significantly reduced the production of exoenzymes, which constitutes the core of the Ps.auerginosa arsenal. Significantly, sotolon reduced the production of protease, elastase and hemolysins by percentages 29%, 28%, and 21% at 25 µg/mL, respectively, and by percentages 71%, 87%, and 84% at 50 µg/mL, respectively (Figure 2B–D). Furthermore, sotolon effectively reduced the ability of the *P. aeruginosa* PAO1 strain to resist oxidative stress. The zone of inhibition of growth of PAO1 by hydrogen peroxide increased significantly in the presence of sotolon by 24% and 49% at 25 µg/mL and 50 µg/mL, respectively (Figure 2E).

### 2.4. Sotolon Downregulated the P. aeruginosa QS Genes

**Figure 3:**
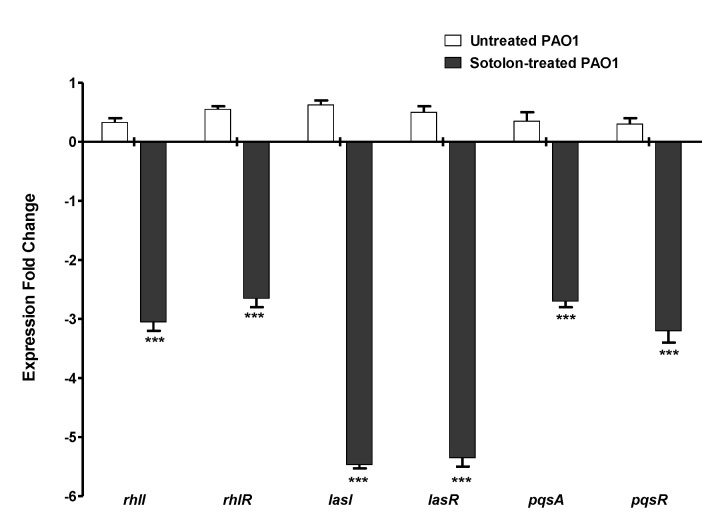
Sotolon downregulated *P. aeruginosa* QS genes. RNA was isolated from PAO1 cultures treated and untreated with sotolon in sub-MIC (50 µg/mL) and was quantified by qRT-PCR and changes in the expression of each QS gene were normalized in relation to the mean critical threshold values of housekeeping gene rpoD. The data shown are the mean ± standard errors from three experiments. *p* < 0.05 was considered significant using one-way ANOVA test. Sotolon significantly reduced the expression of all tested genes (*p* < 0.0001). *** = *p* < 0.001.

The QS genes’ expressions were evaluated in control PAO1 and in sotolon treated bacteria by 2^−∆∆Ct^ method (Figure 3). Sotolon could significantly interfere with the expression of rhlI, rhlR, lasI, lasR, pqsA and pqsR genes that encode the autoinducers and their receptors in *P. aeruginosa*. Meanwhile, sotolon significantly reduced the expression of rhlI, rhlR, pqsA, and pqsR genes by 2- to 3-fold; it downregulated lasI and lasR genes by about 6-fold.

### 2.5. Sotolon Hindered the P. aeruginosa QS Receptors

**Figure 4:**
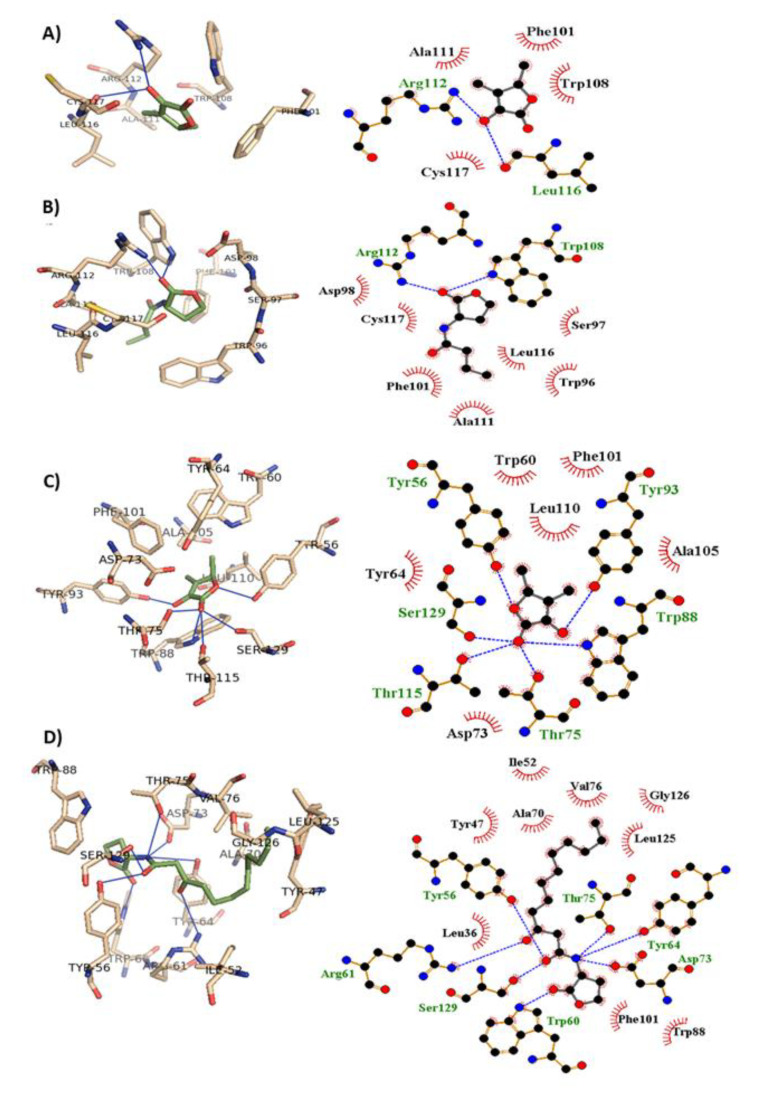
The molecular docking sotolon on *P. aeruginosa* QS receptors. (**A**) Sotolon and (**B**) the natural ligand into the active site of LasR receptor, 3D representation (left) and 2D Schematic interaction (right). (**C**) Sotolon and (**D**) C4-BHL into the active site of RhlR receptor, 3D representation (left) and 2D Schematic interaction (right).

The binding mode of sotolon with lasR and rhlR receptors was revealed from the performed molecular docking study. The binding interactions of sotolon with the lasR receptor and rhlR receptor are shown in Figure 4. The autodock scores for sotolon, the natural ligand along with the interacting residues are presented in Table 1 and Table 2. Sotolon could bind to lasR and rhlR receptors by hydrophobic interaction and hydrogen bonding. The autodock scores revealed that the binding affinity to sotolon was more or less similar to the natural ligands, indicating a strong capability to inhibit quorum sensing. Both the inducer and the inhibitor has nearly the same interaction, however the inhibitor is unable to induce the correct formation of the hydrophobic core of receptor. This conformational change is important for the activity. The stolon lacks the hydrophobic tail and cannot stabilize the conformational change of the protein core [2,38].

### 2.6. Sotolon Protected Mice from P. aeruginosa

**Figure 5:**
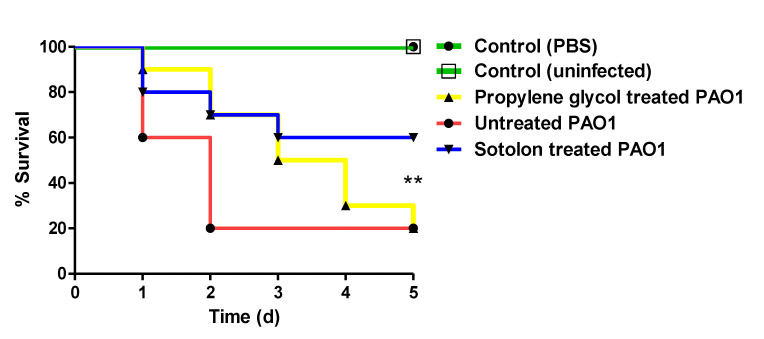
Sotolon in-vivo protection from *P. aeruginosa*. Five groups composed of 10 mice were used: two negative control groups either uninfected or injected with sterile PBS, 2 positive control groups were injected with propylene glycol treated PAO1 or untreated PAO1, and the last group were injected with sotolon in sub-MIC treated PAO1. Mice survival was observed, plotted using the Kaplan–Meier method, and significance (*p* < 0.05) was calculated using a Log-rank test, GraphPad Prism 8. While no deaths were observed in the two negative controls, only 20% of mice were survived in positive control groups. Sotolon conferred 50% protection, as 6 mice injected with sotolon treated PAO1 were survived (Log rank test for trend *p* = 0.0019). ** = *p* < 0.01.

Sotolon protection activity against *P. aeruginosa* pathogenesis was in-vivo evaluated (Figure 5). The survival of mice was monitored in five groups (in each ten mice) for five days. The Kaplan–Meier method was used, and a Log-rank test was performed to calculate significance of sotolon protection from PAO1. All mice survived in the negative control groups, while 80% (8 out of 10) of mice died in positive control groups in which the mice were intraperitoneally injected with propylene glycol treated PAO1 or untreated PAO1. On the other side, sotolon protected 6 mice, conferring 50% protection in comparison to positive control groups. This means that sotolon decreased the PAO1 capacity to kill mice where *p* = 0.0019 by Log rank test for trend.

## 3. Discussion

*P. aeruginosa* is an opportunistic human nosocomial pathogen that shows remarkable resistance to many classes of antibiotics [39]. *P. aeruginosa* can cause infections by an arsenal of quorum sensing controlled virulence factors that enable it to establish and maintain chronic infections. These virulence factors include elastase, proteases, hemolysin, pyocyanin and secondary metabolites in addition to bacterial adhesion and biofilm formation. Anti-virulence agents could be of value in treating pseudomonal infections by disarming its virulence, representing a promising alternative strategy from antibiotics [1,40,41]. In this study, the potential of sotolon as a natural furanone compound to act as an anti-virulence candidate was investigated. Natural and synthetic halogenated furanones were previously found to interfere in virulence and quorum sensing in *P. aeruginosa* [34]. Moreover, a natural furanone ascorbic acid was found to diminish the QS and virulence factors in *P. aeruginosa* [42]. In our previous study, sotolon could inhibit virulence of *Serratia marcescens* in-vitro [37]. Therefore, in this study, we aimed to investigate whether sotolon can show both in-vitro and in-vivo anti-QS and anti-virulence activity against *P. aeruginosa*.

The minimum inhibitory concentration of sotolon was determined, and then the anti-virulence activity of sotolon was assessed at sub-inhibitory concentrations to exclude the effect of sotolon on bacterial growth. Bacterial biofilms are associated with antibacterial resistance and contribute to serious infections, and their formation is controlled by the QS system [11,43]. Sotolon could significantly reduce biofilm formation by *P. aeruginosa*. Pyocyanin enables *P. aeruginosa* to penetrate the membranes of the host cell, and due to its redox-active properties, pyocyanin can interfere with various cell functions resulting in host cell damage [44]. Considerably, sotolon lowered the characteristic bluish-green pigment pyocyanin.

*P. aeruginosa* possesses an arsenal of virulence factors that compromises a diversity of weapons, exoenzymes among all these weapons are on the front attack lines. Proteases, hemolysin and elastase are strongly related to the invasiveness and pathogenesis of *P. aeruginosa*. Proteases target antibodies responsible for protection of mucous membranes. In addition, proteases can damage the tight junction between host epithelial cells, resulting in invasion of host tissues and their damage [45,46,47,48]. Hemolysins causes inflammation and damage to the host tissues and interferes with neutrophil activity [29]. On the other hand, elastase aids in infections by degradation of the host tissue elastin, and decomposition of immunoglobulins [49]. In particular, these three exoenzymes facilitate the spread of *P. aeruginosa*, which results in difficulty of its eradication by the host immunity [3,45,50]. Sotolon significantly diminished the activities of protease, hemolysin and elastase. The immune system efficiency in the eradication of invading microbes depends on their degradation inside the phagosome of antigen-presenting cells to be presented to other immune cells, which is mainly owed to the produced oxidative stress inside phagosomes [45,51,52,53]. Through its role, the serious invading microbe *P. aeruginosa* resists these oxidative stresses [45]. QS affects the tolerance to oxidative stress in *P. aeruginosa* by enhancing its ability to block phagocytosis and oxygen free radical-mediated intracellular killing in macrophages [1,31]. Resistance of *P. aeruginosa* to oxidative stress in the presence of sotolon was reduced significantly.

There are three main QS systems which orchestrate the *P. aeruginosa* pathogenesis. Two LuxI/LuxR QS systems, LuxI homologs LasI and RhlI synthesize C12-homoserine lactone and butanoyl homoserine lactone autoinducers that bind to LuxR homologs LasR and RhlR receptors, respectively [2,10,54]. Moreover, there is LuxR orphan homolog “QscR”, which does not have its own autoinducer but binds to LasI autoinducers [55]. Beside LuxI/LuxR types, there is a non-LuxI/LuxR PQS system [56]. In this study, we evaluated the influence of sotolon on the QS encoding genes and its binding affinity to QS receptors. Interestingly, sotolon markedly down-regulated all of the QS encoding genes of the main three QS systems LasI/LasR, RhlI/RhlR and PqsI/PqsR. In-silico studies reveal that sotolon binds to LasR and RhlR receptors by hydrogen bonding and hydrophobic interactions. The high docking score indicates a high binding affinity to the receptors, making sotolon a potential antagonist that interferes with the binding of the natural ligands to LasR and RhlR receptors.

The phenotypic and genotypic results of sotolon against virulence of *P. aeruginosa* were supported by evaluation of the capacity of sotolon to protect mice from its pathogenesis. In accordance with in-vitro and in-silico results, sotolon in sub-MIC protected 50% of mice from *P. aeruginosa* pathogenesis in-vivo. Finally, sotolon showed a marked mitigation in *P. aeruginosa* virulence, and efficient hindrance to QS systems. It is worth mentioning that our findings regarding the sotolon anti-virulence activities are in great compliance with those obtained by other furanone derivative ascorbic acid anti-virulence activities [42]. El-Mowafy et al., showed that the MIC of sodium ascorbate to *P. aeruginosa* PAO1was 100 mg/mL; meanwhile, the MIC of sotolon to the same strain was 200 µg/mL. While sotolon in 50 µg/mL inhibited the biofilm formation (60%), as well as production of pyocyanin (56.5%), protease (71%), hemolysins (83.5%), and elastase (87%); sodium ascorbate reduced the same virulence factors in a higher concentration of 12.5 mg/mL (64%, 55%, 86%, 85%, and 78%, respectively). Furthermore, sotolon (50 µg/mL) was able to down-regulate the QS encoding genes at comparable rates to sodium ascorbate (12.5 mg/mL). Based on these findings, the sotolon curtailed the *P. aeruginosa* virulence factors in lower concentrations and at a higher efficiency than sodium ascorbate. This means that natural furanone derivatives could serve as safe, competent, and target specific anti-QS and anti-virulence agents, and we potentially propose such chemical moiety as a pharmacophore for future anti-QS and anti-virulence agents against *P. aeruginosa* and other bacteria.

## 4. Materials and Methods

### 4.1. Bacterial Strain, Media and Chemicals

The used *P. aeruginosa* PAO1 strain was donated by the Department of Microbiology, Faculty of Pharmacy, Mansoura University. Microbiological media, Mueller Hinton (MH) broth, Tryptone soya broth (TSB) and agar, and Luria-Bertani (LB) broth and agar were purchased from Oxoid (Hampshire, UK). All chemicals were of pharmaceutical grade. Sotolon, and elastin Congo red dye were obtained from Sigma (St. Louis, MO, USA).

To show the anti-virulence activities of sotolon, a single pure colony of PAO1 was selected and grown OVN with shaking to O.D600 0.4, which equivalates to approximately 1 × 10^6^ CFU/mL. Then, the bacterial suspensions were added to suitable media (according to each experiment) with sotolon in sub-MIC, or control without sotolon. Our results were normalized in all of the experiments by using a fixed bacterial inoculum size (1 × 10^6^ CFU/mL).

According to a sotolon supplier, it was provided as 10 wt.% in propylene glycol. To exclude any effect of propylene glycol, we treated the PAO1 strain with propylene glycol in equivalent concentrations to those used with sotolon (1/4 or 1/8 MIC). It is worthy to mention that propylene glycol in the used concentrations did not have any significant influence on bacterial growth or bacterial virulence (data not shown).

### 4.2. Minimum Inhibitory Concentration (MIC) Determination

The broth microdilution method, according to the Clinical and Laboratory Standards Institute (CLSI, 2014), was employed to detect the MIC of sotolon against PAO1 strains [2]. Briefly, sotolon dilutions in Mueller–Hinton (MH) broth were prepared and added in 100 µL aliquots to a 96-wells microtiter plate. An overnight culture of PAO1 strain in MH broth was suspended in sterile saline, and the turbidity was adjusted to be equivalent to a 0.5 Mac Farland standard. The saline suspension was diluted at 1:100 in MH broth to prepare a suspension with a cell density of approximately 1 × 10^6^ CFU/mL. The bacterial suspension was delivered in aliquots of 100 µL to all the wells with sotolon dilutions and incubated overnight at 37 °C. The MIC was calculated as the lowest concentration of sotolon that inhibited the visible growth of PAO1 strains.

### 4.3. Assessment of Biofilm Inhibition

The sotolon ability to reduce the biofilm formation was quantified as previously described [2,14,48]. Briefly, PAO1 overnight cultures in TSB were prepared and diluted with TSB to an optical density OD600 nm of 0.4. Aliquots (10 µL) of the optically adjusted PAO1 suspensions were added each to 1 mL of fresh TSB with and without 25 µg/mL and 50 µg/mL of sotolon. Aliquots (100 µL) of TSB with and without sotolon were added into a 96-well microtiter plate. The planktonic cells were aspirated after incubation at 37 °C overnight, and the wells were washed thrice and left to dry. The adherent bacterial cells were fixed for 25 min with methanol, followed by staining with crystal violet (1%) for another 20 min. The excessive dye was washed, the adhered dye was dissolved in glacial acetic acid (33%), and the absorbance was measured at 590 nm using a BiotekSpectrofluorometer (Biotek, Winooski, VT, USA). The test was made in triplicate and the absorbance of sotolon treated PAO1 was shown as mean ± standard error of percentage change from untreated controls.

### 4.4. Assessment of Pyocyanin Production

The ability of sotolon to reduce pyocyanin production was estimated as described earlier [2,57]. PAO1 overnight cultures were prepared and diluted in LB broth to 600 nm optical density (0.4). One ml LB broth tubes containing sotolon (25 or 50 µg/mL) or without sotolon were inoculated with 10 μL of the bacterial suspensions, and incubated at 37 °C for 48 h. The tubes were centrifuged at 9500× *g* for 10 min, and the pyocyanin in the supernatant was spectrophotometrically measured at 691nm by a Biotek Spectrofluorometer (Biotek, Winooski, VT, USA). The test was conducted in triplicate, and the stain absorbance in the presence of sotolon was expressed as mean ± standard error of percentage change from untreated PAO1 controls.

### 4.5. Evaluation of Protease Production

In order to evaluate the inhibitory effect of sotolon on protease activity, the skim milk agar method was employed as described [2,30]. PAO1 overnight cultures in LB broth with or without sotolon (25 or 50 µg/mL) were centrifuged at 9500× *g* for 20 min. Aliquots (100 µL) of supernatants were added to the wells made in 5% skim milk agar plates, incubated at 37 °C overnight, and the formed clear zones around the wells were measured. The test was performed in triplicate, the obtained results were presented as mean ± standard error of percentage change from untreated controls.

### 4.6. Evaluation of Elastase Production

Elastase activity of the PAO1 strain was assessed in the sotolon presence (25 or 50 µg/mL) and absence as described previously [2]. The supernatants prepared for skim milk assay were utilized for assessment of elastase production. Elastin Congo red reagent was preprepared by addition of 10 mg of elastin Congo red to 0.5 mL of buffer composed of Tris pH 7.2 (100 mM) and CaCl2 (1 mM). Supernatant aliquots (0.5 mL) were added to elastin Congo red reagent and incubated with agitation at 37 °C for 6 h. Then, the insoluble elastin Congo red was removed by centrifugation. The absorbance of the released Congo red dye by the action of elastase enzymes was measured at 495 nm by Biotek Spectrofluorometer (Biotek, Winooski, VT, USA). The test was performed in triplicate, and the absorbance in the presence of sotolon was shown as mean ± standard error of percentage change from untreated PAO1 controls.

### 4.7. Evaluation of Hemolysins Production

The hemolytic activity in the supernatants of sotolon in sub-MIC treated and untreated PAO1 strains was evaluated, as described earlier [2,13,30]. Briefly, optically adjusted PAO1 cultures treated or untreated with sotolon in sub-MIC were centrifuged, and 500 µL of supernatants were mixed with fresh 800 µL erythrocytes 2% suspension in saline. The mixtures were incubated at 37 °C for 2h. A positive control representing complete hemolysis was obtained by addition of Sodium dodecyl sulphate (SDS) to erythrocytes suspension, and negative control was obtained by incubation of erythrocytes in LB broth under the same circumstances. The hemoglobin released from erythrocytes was measured at 540 nm by Biotek Spectrofluorometer (Biotek, Winooski, VT, USA) in the supernatants obtained by centrifugation of the mixtures at 2600× *g* for 5 min at 4 °C. The experiment was conducted in triplicate, and the hemolysis of sotolon treated cultures were presented as mean ± standard error of percentages compared to those obtained from untreated control cultures.

### 4.8. Evaluation of Sensitivity to Oxidative Stress

The ability of sotolon to reduce pyocyanin-mediated resistance to oxidative stress was estimated by the disk assay method [2,14]. Overnight cultures of the tested bacterial strains in TSB were prepared, and aliquots of 100 μL were uniformly spread on the surface of TSA plates with 2% agar with and without sotolon in sub-MIC. Sterile filter paper disks (6 mm diameter) were put on the surface of TSA plates, and the disks were loaded with 10 μL of 1.5% hydrogen peroxide. The plates were kept at 37 °C for 24 h, and the diameters of the inhibition zones were measured. The test was made in triplicate. The test was performed in triplicate, the obtained results were presented as mean ± standard error of percentage change from untreated controls.

### 4.9. Evaluation of Expression of QS Encoding Genes

The ability of sotolon to interfere with the expression of QS genes was assessed by RNA extraction from PAO1 cultures treated and untreated with sotolon (50 µg/mL) [2,30]. Briefly, sotolon-treated and untreated PAO1 cultures were collected by centrifugation (7000 rpm for 10 min at 4 °C). The formed pellets were re-suspended in Tris-EDTA buffer (100 μL) provided with lysozyme and kept at 25 °C for 5 min. Bacterial pellets were lysed by RNA lysis buffer, and total RNA was isolated and purified using GeneJET RNA Purification Kit protocol (Thermoscientific, Waltham, MA, USA). DNase was used to remove residual chromosomal DNA. Finally, RNA concentrations were measured by NanoDrop ND-1000 spectrophotometer and stored at −70 °C until use.

The primers used to evaluate the relative QS gene expression levels in PAO1 strains by the qRT-PCR were previously listed [42]. The cDNA was obtained using a cDNA Reverse Transcriptase kit (Applied Biosystem, Beverly, MA, USA) that was amplified by the PCR Master Kit Syber Green I (Fermentas), by employing the Step One instrument (Applied Biosystem, Beverly, MA, USA). The PCR amplification steps were 10 min/95 °C followed by 40 repeated cycles of 20 s/95 °C, 20 s/62 °C, and 65 s/72 °C. The housekeeping *ropD* gene was used as the reference gene, to normalize the expression of QS genes, and the relative gene expression was calculated by the comparative threshold cycle (ΔΔCt) method, as described earlier [2,47]. The experiment was done in triplicate, and the expression of sotolon treated bacterial genes were presented as mean ± standard error of fold change from untreated PAO1 controls.

### 4.10. In-Silico Study of Sotolon Binding to QS Receptors

The sotolon molecular interactions with the QS receptors LasR and rhlR was investigated by docking analysis. The crystal structures of *P. aeruginosa* ligand binding domain models of rhlR and LasR receptors were obtained from the protein model portal (ID: P54292.1) and Protein Data Bank (PDB ID: 2UV0), respectively [38]. The capability of sotolon and the natural ligands butanoyl homoserine lactone (C4-HSL) and 3-oxo-dodecanoylhomoserine lactone (C12-HSL), to interact with the active sites of rhlR and LasR receptors, was determined as previously described [3]. Briefly, for both ligands and receptors, the AutoDockTools software [42] was used to produce the pdbqt files after assigning the atomic partial charges using the Gasteiger method [43]. The docking settings, including the num modes of 10, exhaustiveness of 12 and grid boxes center (x = 23.02, y = 15.86 and z = 80.07) with size (x = 13, y = 13, z = 13) and center (x = 5.43, y = 64.84 and z = 40.27) with size (x = 13, y = 13, z = 13) were used for autodock vina [34] docking into the active site for LasR and rhlR, respectively.

### 4.11. Sotolon In-Vivo Protection

The sotolon protective activities on *P. aeruginosa* PAO1 pathogenesis were assessed by the mice survival model, as described previously [2,30,52]. The PAO1 cultures with or without sotolon (50 µg/mL) were adjusted to 1 × 10^6^ CFU/mL (OD600 of 0.4) in phosphate buffered saline (PBS), and in broth with propylene glycol in the same concentrations that have been applied as a solvent for sotolon. Five groups, each containing 10 healthy female albino mice with similar weights, were used. There were two negative control groups: one group was left uninoculated, and the other group was intraperitoneally injected with sterile PBS (100 μL). There were also two positive control groups: one injected with 100 μL of propylene glycol (used solvent of sotolon) treated PAO1, and the other was injected with 100 μL of untreated PAO1. The last group was injected with 100 μL of sotolon-treated PAO1. The survival of mice was observed daily for five successive days, and the deaths were recorded.

### 4.12. Statistical Analysis

One way ANOVA test, Graph Pad Prism 8 was employed for statistical analysis of the inhibitory activities of sotolon on *P. aeruginosa* virulence factors, and this was followed by post-test Dunnett’s Multiple Comparison. Statistical significance was considered when *p* values < 0.05.

## 5. Conclusions

Targeting QS and bacterial virulence is a promising strategy to overcome the endless development of bacterial resistance towards the antibiotics. The merits of this strategy can be greatly enhanced by using safe compounds, such as those extracted from plants or other safe natural sources. In this study, we evaluated the anti-QS and anti-virulence activities of the sotolon aromatic furanone compound which gives fenugreek its smell. Significantly, sotolon diminished the *P. aeruginosa* pathogenesis in-vitro and in-vivo. Furthermore, sotolon downregulated the *P. aeruginosa* QS encoding genes, and showed considerable capability to hinder the QS receptors. In this study, we are introducing the sotolon by its furanone moiety as a natural virulence mitigating agent in *P. aeruginosa*, that can be used as an alternative or as an adjuvant to traditional antimicrobial therapies. Our preliminary findings pave the way for further pharmacological and pharmaceutical studies, which can result in safe use of sotolon in management of bacterial infections beside traditional antimicrobials.

## Figures and Tables

**Table 1:** The binding mode of each ligand with the different residues inside the active site of lasR.

Ligand	H-Bonding	Hydrophobic Interaction	Autodock Score
**Sotolon**	Tyr 56, Thr 75, Trp 88, Tyr 93, Thr 115, Ser 129	Tyr 56, Trp 60, Tyr 64, Asp 73, Thr 75, Trp 88, Tyr 93, Phe 101, Ala 105, Leu 110, Thr 115, Ser 129	−6.3
**C12-HSL**	Tyr 56, Arg 61, Tyr 64, Asp 73, Thr 75, Trp 60, Ser 129	Leu 36, Tyr 47, Leu 52, Tyr 56, Trp 60, Arg 61, Tyr 64, Ala 70, Asp 73, Thr 75, Val 76, Trp 88, Phe 101, Leu 125, Gly 126, Ser 129	−8.3

**Table 2:** The binding mode of each ligand with the different residues inside the active site of rhlR.

Ligand	H-Bonding	Hydrophobic Interaction	Autodock Score
**Sotolon**	Leu116, Arg 112	Phe 101, Trp 108, Ala 111, Leu116, Cys 117, Arg 112	−5.6
**C4-BHL**	Trp 108, Arg 112	Ser 97, Trp 96, Asp 98, Phe 101, Trp 108, Ala 111, Leu116, Cys 117, Arg 112	−6.1

## Data Availability

The data presented in this study are available on request from the corresponding author.
